# Macrofungal sporocarp community in the lichen Scots pine forests

**DOI:** 10.1515/biol-2022-0973

**Published:** 2024-09-23

**Authors:** Barbara Grzesiak, Michał Hubert Węgrzyn, Agnieszka Turowska, Magdalena Twarużek

**Affiliations:** Department of Environmental Biology, Faculty of Natural Sciences, Kazimierz Wielki University, Ossolińskich 12, 85-093, Bydgoszcz, Poland; Prof. Z. Czeppe Laboratory of Polar Research, Institute of Botany, Faculty of Biology, Jagiellonian University, Gronostajowa 3, 30-387, Kraków, Poland; Bory Tucholskie National Park, Długa 33, 89-606, Charzykowy, Poland; Department of Physiology and Toxicology, Faculty of Natural Sciences, Kazimierz Wielki University, Chodkiewicza 30, 85-064, Bydgoszcz, Poland

**Keywords:** macrofungi, *Cladonio-Pinetum* community, natura 2000 habitat, national park, Tuchola forest, active protection

## Abstract

The lichen-rich pine forests of *Cladonio-Pinetum* represent special habitats protected under the Natura 2000 scheme. A rapid decline in their density has been observed in recent years. Macrofungi are an important component of the community and sensitive bioindicators; therefore, observations of the response of their sporocarps can be used to draw conclusions about changes induced in habitats. In our study, we tried to detect differences in species richness, composition, and biomass of the ectomycorrhizal fungal communities in areas rich in lichens indicating the good state of the community, and rich in bryophytes signaling its progressive degradation. The influence of precipitation and temperature on species richness and biomass was checked, and the possibility of using the sporocarps-based method to assess the effectiveness of active protection treatments, which are a form of partial protection carried out to maintain specific natural habitats. For detailed studies, six plots were selected from which sporocarps were collected, dried and weighed. A total of 1,575 sporocarps, representing 49 taxa were collected. A higher number of taxa (39) in lichen-rich plots were found. In bryophyte-rich areas, 27 taxa were recorded. The total yield was 13,151 g dry weight ha^−1^. In lichen-rich plots, the biomass was almost half lower.

## Graphical abstract

**Figure:**
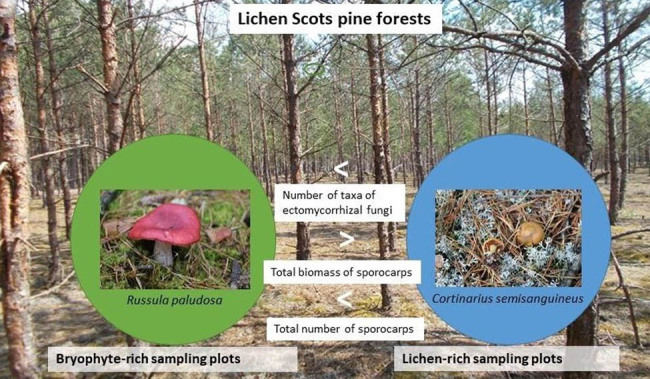

## 1 Introduction

Central European lichen Scots pine forests (which belong to communities of *Cladonio-Pinetum* association) are protected by European Union’s legislation (Natura 2000 habitat, code 91T0). They represent a significant forest ecosystem due to their diverse lichen species, especially of the genus *Cladonia* [1,2]. Several of them are legally protected in Poland and listed in the annex of the Habitats Directive of the European Union [3]. The lichen Scots pine forests occur in the driest and most nutrient-poor areas, and because of that, they have lower productivity compared to other pine forests on mineral soils [4,5]. Due to its oligotrophic characters, the *Cladonio-Pinetum* community is not characterized by rather low species biodiversity of lichens, but there are many rare species, occurring only in this type of habitat [1]. It occurs not only in Central Europe, particularly in the Czech Republic [1], Germany [6], Lithuania [7], Slovakia [1], and Poland [1,2] but also in the Netherlands [8], Finland [4], Norway, Latvia, and Estonia [9].

Since the late twentieth century, the lichen Scots pine forests are rapidly disappearing not only in Poland, but also throughout Europe [2,10,11,12,13]. The main causes of this phenomenon are strong eutrophication of the substratum [2,10–13] and a gradual increase in carbon sequestration in the forest soils [2]. This plant community is probably anthropogenic, semi-natural, and associated with the use of forests in the past – e.g., cattle grazing, litter raking, and extensive forestry management. These treatments contributed to a decrease in soil nutrients, which in turn contributed to the long-term stability of these communities [2]. The condition of the *Cladonio-Pinetum* forest habitat is evidenced by the undergrowth rich with lichens [18]. An increased proportion of bryophytes in the undergrowth suggests transformation into more fertile habitats and its degradation [2,19]. Many authors are looking for answers to the question of what factors influence the disappearance of these valuable communities [13].

Macrofungi are an important component of the lichen Scots pine forests in terms of structure and functionality – as symbionts of pine and decomposers, they can be regarded as true specialists in sandy, oligotrophic pine forests [20,21]. Ectomycorrhizal fungi are crucial in the functioning of forest ecosystems with *Pinus sylvestris* [22], also *Cladonio-Pinetum* community. These fungi represent a large part of the biodiversity in boreal forests. They depend on carbohydrates from their host trees and are vital for forest production, as uptake of nutrients and water by the trees is mediated by the ectomycorrhizal symbiosis [23]. Based on their sporocarps, we can draw conclusions about the changes that take place in the habitat [20,24,25,26]. Sporocarp production has been the focus of many studies, and numerous variables influencing fungal production have been identified, e.g., precipitation, temperature, and stand structure characteristics [27]. The very specific habitat requirements of fungi make them well suited as indicators for selecting conservation areas and monitoring their status [28]. Studies based on the analysis of sporocarps provide a lot of data and allow us to show the diversity of fungal communities and red-listed species. Sporocarp fungal communities also respond to environmental factors. They are a good complement to modern methods, such as metabarcoding and high-throughput DNA soil sequencing, which do not always demonstrate the all species richness of mycobiota and indicator species, and are more expensive than traditional studies [29,30].

The literature indicates that the type of undergrowth has a significant impact on the biodiversity and biomass of fungi. Genevieve et al. [31] found that covering with moss results in a decrease in fungal richness, and the decline in fungal richness was also observed in older stand. Fungi living in moss covered tree stand, would have considerable potential to degrade carbon substrates contained in moss residues. Mosses can influence soil conditions by keeping water levels high and slowing nutrient circulation. For lichen cover, no information was found explaining how it may directly influence fungal richness, but Genevieve et al. [31] found that the type of stand where there is high abundance of lichen is not favorable for fungi. In turn, studies from Finland [20], where there are well-preserved patches of lichens in the dry oligotrophic Scots pine habitat, indicate that during 4 years of studies, 207 species of fungi were found there, mainly from the genera *Cortinarius, Lactarius, Russula,* and *Suillus*, which constituted a large share in the biomass of all sporocarps. Based on these observations from different regions, we decided to investigate whether the type of undergrowth affects the species richness, composition, and biomass of ectomycorrhizal fungi. Our goal was to test it with a new field study in particularly well-preserved lichen pine forest in Poland. We compared two types of plots characterized by different degrees of preservation of the community – with a high share of lichens in the undergrowth, indicating a good condition of the community, and with a high share of bryophytes, indicating the progressive degradation of the community. According to Tomao et al. [27], precipitation, temperature, and stand structure characteristics (e.g., tree species composition, stand age, and stand density) influence fungal yields. Second objective was to determine how climate factors, such as temperature and precipitation, affect the biomass and species diversity of ectomycorrhizal fungi recorded in plots rich in lichens and bryophytes. Additional objective was to investigate whether the condition of the *Cladonio-Pinetum* habitat after active protection treatments could be determined based on a sporocarps inventory. Using fungal sporocarps to assess habitat, increases the robustness of the assessment because fungi are very sensitive indicators of habitat changes [20,24,25,26,28]. Changes in habitat conditions result not only in changes in the species composition of fungi, but also in changes in the abundance of sporocarps. Often, changes in the number of sporocarps produced precede changes in the mycelium, and are the first signal of its reaction [32].

We hypothesize that: (1) the species richness and sporocarps biomass production be significantly different between lichen-rich and bryophyte-rich sampling plots and (2) there are significant differences in the phenology of ectomycorrhizal fungi between the two types of plots.

## 2 Materials and methods

### 2.1 Study area

**Figure 1:**
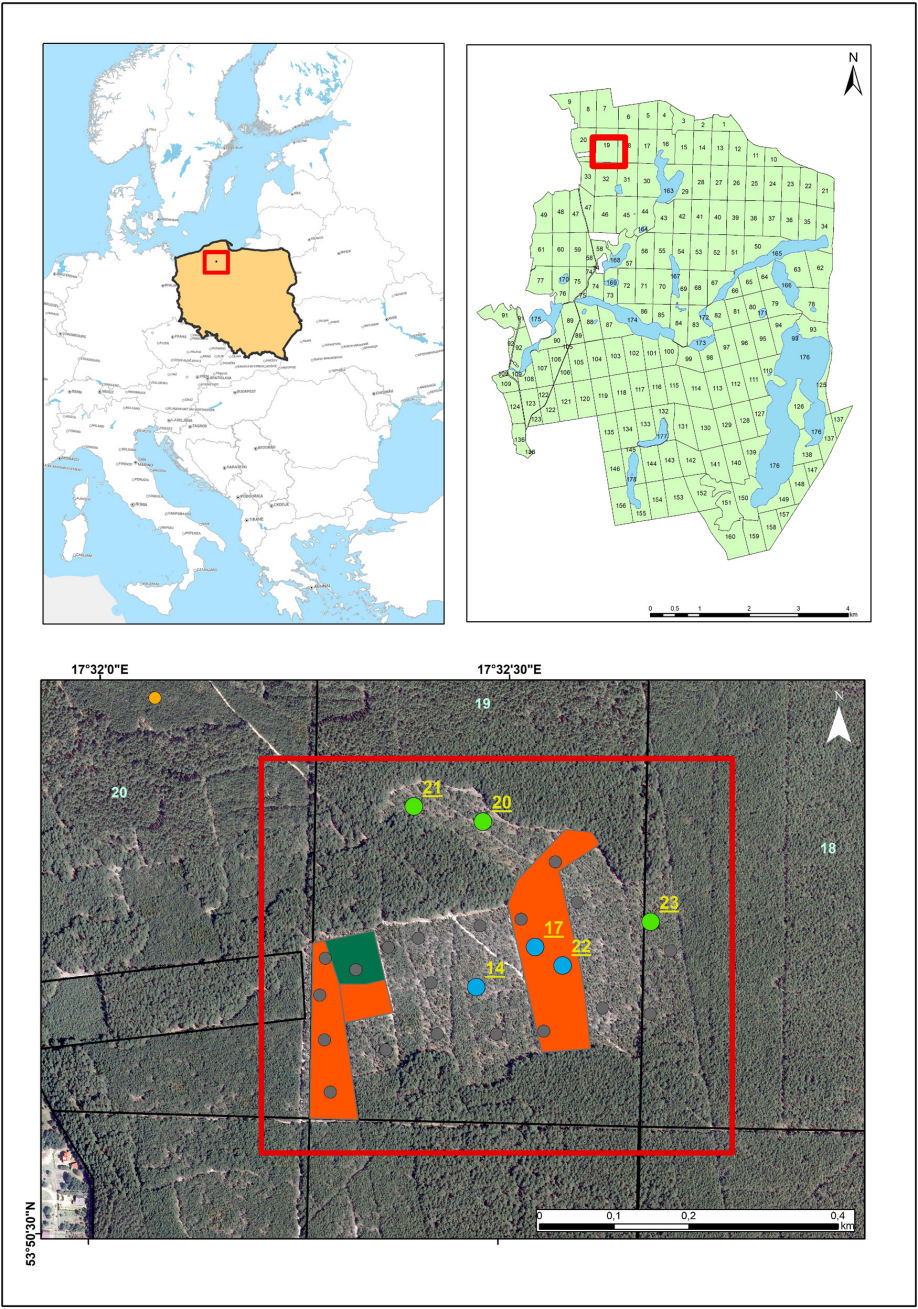
Map of the location of the study area in the Bory Tucholskie National Park (Poland) (red line: entire area subjected to active protection treatments, thinning cuts, and removal of dead wood; orange area: forest litter raking; dark green area: removing bryophytes; light blue numbers: forest section; 25 sampling plots [light grey dots]; sampling plots selected for study – blue dots: numbers 14, 17, 22 [yellow] – lichen-rich sampling plots; green dots: numbers 20, 21, 23 [yellow] – bryophyte-rich sampling plots; light orange dot in forest section number 20: control plot).

One of the best-preserved patches of the *Cladonio-Pinetum* community can be found in the north of Poland, including the Bory Tucholskie National Park (north-west Poland), but there are also habitats with a disappearing layer of lichens in the undergrowth. The National Park is located in north-west Poland (Figure 1), on sands deposited on glacial tills formed during the last glaciation period [33], and spans a total area of 4,613.04 ha. Dry pine forests in the midland of *Cladonio-Pinetum* occupy 20.28% of terrestrial communities, cover 930.58 ha, and are designated as protection objects [34,35].

Due to the increasing disappearance of lichen Scots pine forest in the Bory Tucholskie National Park, in 2017, the study area of 12,609 ha was selected, and active conservation treatments were taken, including, among others: thinning the tree stand (canopy cover – 0.6, 60%), removing of organic matter in the form of the dead wood lying in the undergrowth, and raking the litter in the experimental area (Figure 1) [2]. The objective of active conservation measures was to reduce shading and eutrophication, and as a consequence, transforming the undergrowth rich in bryophytes into an undergrowth rich in lichens [1,2,11,12,13,36].

### 2.2 Sporocarps sampling

Out of 25 circular sampling plots (area 100 m, radius 5.64 m) where mycological studies were conducted in 2021, 6 were selected for detailed biomass analysis. A list of all fungi species found in 2021 in 25 sampling plots (and one control plot) is attached as supplementary material (Appendix A, List A.1). The most representative plots in terms of type of undergrowth were selected based on the study by Węgrzyn et al. [2], which included phytosociological data, the geographical coordinates, age of three stands (Tables S1 and S2) and a division into two types of habitat preservation. Three of them were characterized by a high participation of bryophyte (above 80%) (nos 20, 21, and 23; total area of approximately 300 m^2^), and three, by a high degree of coverage by terricolous lichen species of the *Cladonia* spp. genus (above 70%) (nos 14, 17, and 22; total area of approximately 300 m^2^). The age of tree stands in the sampling plots was as follows: plot no. 23 was 58 years old, plot nos 17, 20, 21, and 22 were 39 years old, and plot no. 14 was 29 years old [37]. The six plots selected for detailed analyses were subjected to thinning cuts and the removal of dead wood. Within areas 17 and 22, forest litter was raked (Figure 1).

The study in the 25 sampling plots was conducted from May to November 2021, twice a month. Sporocarps within all selected six plots were collected in the early morning hours every 2 weeks. The research material consisted of macroscopic fungi (macrofungi), i.e., those with the size of sporocarps exceeding 1 mm, visible to the naked eye [38,39].

All sporocarps of macrofungi within the sampling plots were identified and counted. In the case of species causing taxonomic difficulties, specimens were brought to the laboratory to confirm field identifications, dried, and included in a voucher collection. They were deposited in the collections of the University of Kazimierz Wielki in Bydgoszcz. Further investigations of unidentified taxa using molecular techniques are in the planning stages.

Further, detailed biomass studies focused only on ectomycorrhizal fungi because these organisms are the most sensitive to the changes taking place in the surrounding habitat, as the number of their sporocarps and biomass may indicate the direction of these changes and the condition of mycorrhizae [32]. Additionally, fungal communities respond differently to environmental drivers, with ectomycorrhizal symbionts and parasites being more related to tree traits than saprotrophs, which were affected mostly by climate and substrate [30]. Many researchers [22,23,40,41,42] conducted biomass research with ectomycorrhizal species; therefore, this group of fungi was selected to make it easier to compare our study with other studies.

Sporocarps of ectomycorrhizal fungi were collected from the sampling plots by twisting them completely; they were premarked, counted, and packed into separate string bags. Further stages of the studies were carried out in the laboratory. Materials brought from the sites were cleaned, segregated into species, and dried at 50–60°C in Niewiadów 970 dryers (330 W power) (Niewiadów S.A.) for 1–2 days. Due to the size of sporocarps, two scales with two different accuracies were used to weigh the exsiccates of fungi: Mesko MS3152 (5 kg; accuracy, 1 g) (Mesko) and MH-Series MH-200 (MH) (200 g; accuracy, 0.01 g). In this type of study, it is recommended to use dry matter, as the water content of sporocarps of different species varies and depends on weather conditions [32]. The fresh weight values for analyses were calculated by multiplying the value of dry matter by 10 [32,38], because the average water content of sporocarps is assumed to be 90%.

Sporocarps were also counted in the sampling plots, because the use of the sporocarps production parameter in parallel with the sporocarps abundance parameter made it possible to avoid the equal treatment of species producing sporocarp that differ significantly in size (biomass) [32].

The material was identified using standard mycological methods. Microscopic characteristics were viewed using a Nikon Eclipse 50i light microscope (Nikon) and an Olympus SZ61 stereoscopic microscope (binocular) (Olympus). Specimens were identified by examining macroscopic and microscopic features, using monographs by Antonín and Noordeloos [43], Bas et al. [44,45,46,47], Bessette et al. [48], Breitenbach and Kränzlin [49,50,51,52], Galli [53], Knudsen and Vesterholt [54], Kränzlin [55,56], and Noordeloos et al. [57].

The nomenclature of basidiomycetous fungi and functional group of fungi were accepted following Knudsen and Vesterholt [54]. The nomenclature of species not included in the study by Knudsen and Vesterholt [54] is in accordance with the Index Fungorum [58]. The threat categories of fungi are given according to Wojewoda and Ławrynowicz [59].

## 3 Results

### 3.1 Species richness of ectomycorrhizal fungi

A total of 49 taxa belonging to 32 species, 1 subspecies, 13 genera, 1 variety, and 2 forms were found in 6 sampling plots. A higher species richness was found in the lichen-rich sampling plots, with 39 taxa, while 27 taxa were recorded in the bryophyte-rich sampling plots (Appendix B, Table B1). In plot number 14, the highest number of taxa among lichen-rich sampling plots was observed, whereas the lowest number was observed in plot number 17. Among the sampling plots with a large share of bryophytes, the highest number of taxa was noted in plot no. 20, and the lowest number was recorded in plot no. 23 (Table 1).

**Table 1:** Species richness, number of sporocarps, and dry biomass of macrofungi in sampling plots

	Bryophyte-rich sampling plots			Lichen-rich sampling plots		
Plot no.	20	23	21	22	17	14
Age of stands (years)	39	58	39	39	39	29
Number of taxa	20	15	17	27	24	28
Number of sporocarps	258	201	130	271	138	423
Dry biomass (g)	144.55	219.56	153.62	105.76	55.27	110.28

Note: The age of tree stands was according to: Bory Tucholskie National Park [37].

### 3.2 Fungal productivity and taxonomical diversity

**Figure 2:**
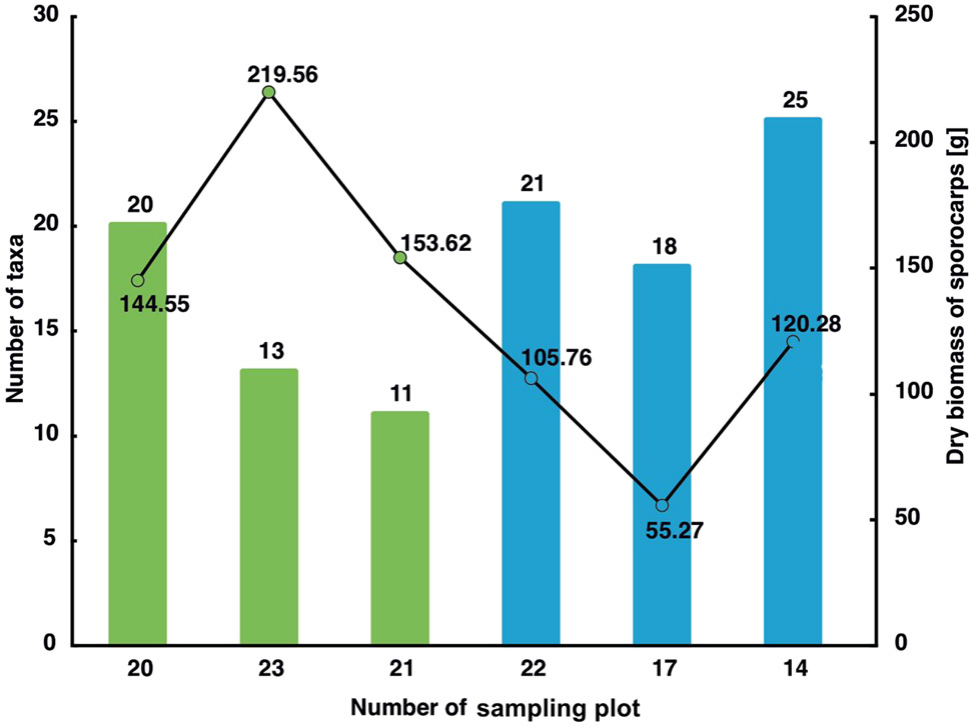
The number of taxa (column graph) and dry biomass of sporocarps (line graph) in the sampling plots (bryophyte-rich plots – green color; lichen-rich plots – blue color).

The total dry biomass of all the harvested sporocarps of ectomycorrhizal fungi was 789.04 g/600 m^2^ (13,151 g dry weight ha^−1^); therefore, the fresh weight was 7.89 kg. Therefore, there is 1.31 g of dry weight of sporocarps of ectomycorrhizal fungi per m² (i.e., 13.15 g of fresh weight). The total biomass of sporocarps of ectomycorrhizal fungi in bryophyte-rich areas was 517.73 g/300 m^2^; in lichen-rich plots, it was almost half lower – 271.31 g/300 m^2^ of dry matter (Appendix B, Table B1). The highest biomass among the sites with mossy undergrowth was recorded in plot 23 (219.56 g dry weight), and the lowest was recorded in plot 20 (144.55 g dry weight). Among the localities with high lichen coverage, plot 14 (120.28 g dry weight) dominated in terms of biomass productivity, while the smallest biomass was found in plot 17 (55.27 g dry weight) (Table 1, Figure 2).

A total of 1,575 individual sporocarps were collected in six sampling plots (Appendix B, Table B1). Cortinariaceae was the most taxa-rich family, with 22 taxa. Not all fungi from the genus *Cortinarius* and *Inocybe* have been identified as species because this genus occasions great taxonomic difficulties. *Amanita* and *Russula* were the next species-rich genera, each containing four species. Bryophyte-rich sampling plots 20, 21, and 23 (approx. 300 m^2^) were characterized by a lower number of sporocarps of ectomycorrhizal fungi (525 sporocarps) than lichen-rich sampling plots of 14, 17, 22 (approx. 300 m^2^), in which 779 sporocarps were collected (Appendix B, Table B1). In the sampling plots with a high percentage of mosses, plot no. 20 dominated in terms of the abundance of sporocarps. The least number of them was recorded in plot no. 21. Among the plots rich in lichens, the plot with the highest number of fruiting bodies was plot no. 14, while the least was found in plot 17 (Table 1, Figure 2).

The analyzed two types of plots differed in terms of species composition (Appendix B, Table B1; Appendix C, Figure C.1). The areas rich in bryophytes were dominated by species of the genera *Xerocomus, Russula* (especially *Russula paludosa*), *Sarcodon, Lactarius, Cortinarius armeniacus,* and also *Cantharellus cibarius*. Species found only in bryophyte-rich plots were *Cortinarius depressus, Cortinarius caperatus, Cortinarius cinnamomeus, Russula vesca, Lactarius necator, Laccaria proxima, Cortinarius mucosus,* and *Cortinarius* sp. 1, 2, and 3. In lichen-rich sampling plots occurred mainly fungi of the genus *Cortinarius*, mainly *Cortinarius croceus* subsp. *croceus, Cortinarius semisanguineus, Cortinarius brunneus, Cortinarius acutus,* as well as *Thelephora terrrestris, Laccaria laccata, Tricholoma terrestris, Tricholoma equestre,* and *Tricholoma albobrunneum* and species of the genus *Amanita*. The species exclusive to lichen-rich areas were *Amanita excelsa* f. *excelsa, Amanita muscaria* var. *muscaria, Amanita rubescens* f. *rubescens, Boletus pinophilus, Coltricia cinnamomea, Cortinarius brunneus, Cortinarius* sp. 4-12, *Inocybe* sp., *Inocybe sambucina, Suillus bovinus, Suillus luteus, Tricholoma albobrunneum, Tricholoma equestre,* and *Tricholoma terreum*.

Analyzing the species composition of both types of study areas and the abundance of sporocarps in two groups (over 20) (Appendix B, Table B1), it appears that the species typical for sites rich in bryophytes were *Cantharellus cibarius, Lactarius rufus, Russula emetica, Russula paludosa, Sarcodon squamosus*, and *Xerocomus badius*. In the lichen-rich plots, the following species occurred more frequently: *Amanita rubescens* f. *rubescens, Cortinarius acutus, Cortinarius brunneus, Cortinarius croceus* subsp. *croceus, Cortinarius semisanguineus, Thelephora terrestris*, and *Tricholoma terreum*.

### 3.3 Dynamics of sporocarp production in individual months of the study season

**Figure 3:**
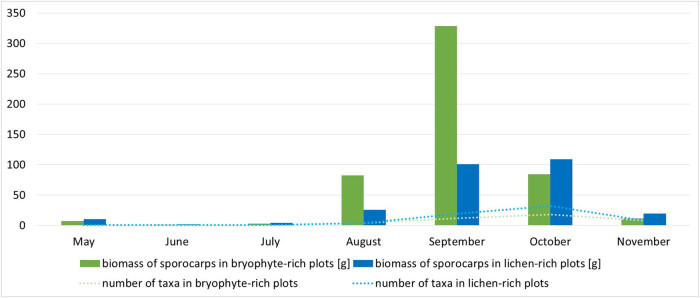
The dynamics of the sporocarps biomass productivity (column chart) of ectomycorrhizal fungi and its species richness in the study season (line graph).

**Figure 4:**
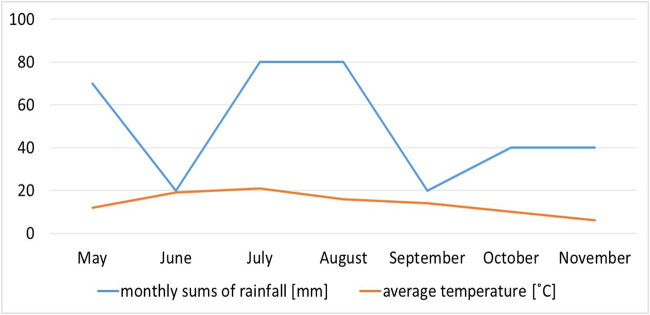
The monthly sums of rainfall (blue line graph) and average temperature (orange line graph) – source of meteorological data: Institute of Meteorology and Water Management in Poland [60].

Analysis of fluctuations in sporocarp biomass productivity in individual months of the survey season shows that in lichen-rich sampling plots, the maximum sporocarps biomass was observed in September, while in bryophyte-rich sampling plots – in October (Figure 3). In areas rich in lichens, a sharp, clear increase in biomass was observed after a period of rainfall (Figure 4). In the case of areas rich in bryophytes, this increase was not so rapid, and the maximum biomass was observed only in October. The fluctuations in the number of taxa during the study season for both types of plots are remarkably similar, and in both cases, the highest number of taxa was recorded in October (Figure 3).

### 3.4 Species of conservation value

During the study, five species included on the *Red list of macrofungi in Poland* [59] were recorded. They were in the category “V” (vulnerable) – *Cortinarius armeniacus*, in the category “R” (rare) – *Cortinarius acutus* and *Cortinarius croceus* subsp. *Croceus,* and in the category “I” (indeterminate) – *Coltricia cinnamomea* and *Tricholoma equestre*. Many species (*Cortinarius caperatus, Boletus pinophilus, Suillus bovinus, Suillus luteus, Russula decolorans, Russula paludosa, Russula vesca*, and *Xerocomus badius*) have been assessed for *The IUCN Red List of Threatened Species* in 2018 and *Boletus edulis –* in 2019. They are listed as “Least Concern” [61].

## 4 Discussion

### 4.1 Species richness and composition of mycobiota lichen Scots pine forest community

The total species richness of ectomycorrhizal fungi in six sampling plots accounts for 46% of all taxa identified from all plots in 2021 (107 taxa) and 83% of all ectomycorrhizal fungi (59 taxa) (Appendix A., List A.1). During the study conducted in the *Cladonio-Pinetum* community between 2014 and 2016 [62], a total of 140 taxa were identified, among which 27 ectomycorrhizal fungi were common. From the area where active protection measures were carried out (forest sections: 18 and 19), of the 71 fungal taxa identified, 27 ectomycorrhizal fungi were common in these studies [63]. Based on the above data, it appears that the number of ectomycorrhizal species from the six areas is high.

Stankevičienė and Kasparavičius [40] and Stankevičienė et al. [41] obtained very similar results regarding the number of ectomycorrhizal basidiomycetes in pine forest from the Lithuania–Poland transboundary region. At nine permanent sampling plots in a 50-year-old pine forest of the *Cladonio-Pinetum* were collected 53 taxa of ectomycorrhizal fungi, of which the majority of them belong to the genera *Cortinarius, Russula, Amanita*, and *Tricholoma*. They were also one of the most frequently found taxa in the lichen Scots pine forests in Bory Tucholskie National Park.

### 4.2 Comparison of mycobiota on sampling plots with different conservation status

Our study provides the first data on fungi in two types of habitat preservation in the lichen Scots pine forest in the area, where active protection treatments were carried out. Habitats with a high share of lichens in the forest undergrowth indicate the good condition of the community, and with a significant proportion of bryophytes in the undergrowth suggest a degeneration of the community patch. In mycological studies [20,21,40,41], habitat and ecological considerations are found, but there is a lack of publications with a breakdown into different degrees of community preservation. Additionally, there are only a few myco-sociological data in *Cladonio-Pinetum* communities from Poland [64], and literature cited there. Our results have shown that these two forms of lichen Scots pine forests differ in terms of the species richness, composition, and biomass of sporocarps.

A higher species richness was found in the lichen-rich sampling plots than in the bryophyte-rich areas. The highest number of taxa was observed in plot number 14, whereas the lowest number was observed in plot number 17. Among the sampling plots with a large share of bryophytes, the highest number of taxa was noted in plot no. 20, and the lowest number was recorded in plot no. 23 (Table 1).

Age of the tree stand may be one of the factors influencing species richness in sampling plots. This fact is confirmed in the study by Genevieve et al. [31]. They observed a decline in operational taxonomic units (OTUs) richness in older stands. Table 1 presents the number of taxa and the age of the stand. As can be seen, the highest number of species was identified in plot 14, where the stand is the youngest (29 years old). The smallest species were found in plot no. 23, where the stand is the oldest (58 years old). Węgrzyn et al. [13] indicated relationships between height of tree stand and the lichen-rich undergrowth in *Cladonio-Pinetum* community. The lichen cover better developed under low stand. In bryophyte-rich plots, tree stands were higher and oldest than in lichen-rich sampling plots [2, Suppl. Table 2]. This resulted in differences in the species richness, composition, and biomass production of ectomycorrhizal fungi communities in the undergrowth layer. Nevertheless, Rudawska et al. [22] arrived at divergent conclusions. They investigated changes in taxonomical and functional structure of the ectomycorrhizal fungal community of Scots pine underpinned by the ageing of the partner trees in the Tuchola Forest. The stands were aged 7–87 years. Of the 46 taxa collected, 13 turned out to be common to our studies. It turned out that they exhibited similar diversity at each age and some of the fungal taxa are gradually replaced with others along the pine age gradient, e.g., *Suillus luteus* prefers young pine forests and *Russula ochroleuca* occurs in old forests. In Scots pine monoculture, the ectomycorrhizal community structure is highly dependent on stand age.

Differences in species composition may be significantly influenced by the type of undergrowth. Moss cover was one of the best predictors of fungal richness. The mosses are well known to be recalcitrant substrates and they can affect conditions growth by maintaining high water level and slowing down the nutrient cycle [31]. The authors observed a decrease in OTUs richness when moss cover was high. In our study, the number of species and sporocarps was higher in lichen-rich sampling plots, which supports their conclusion. Simultaneously, our findings are in opposition to their results, that the type of stand with high abundance of lichen is not favorable for fungi. In areas rich in lichens, there are specialized species, typical of these habitats [21], which do not need high humidity for the development of sporocarps.

Our study also identified differences in biomass of sporocarps in both types of sampling plots. In places rich in bryophytes, the biomass of sporocarps was higher. It is possible that this is due to the fact that the species occurring there has fewer but larger sporocarps, e.g., *Russula* spp. and *Lactarius* spp. In places with a large share of lichens, species with small and numerous sporocarps occur, e.g. *Cortinarius* spp. and *Thelephora* spp. The annual production of dry weight biomass production of terrestrial sporocarps of ectomycorrhizal fungi is 14–68,000 g dry weight ha^−1^ in Scots pine forests [65]. Total annual biomass in our study was 13,151 g dry weight ha^−1^, with 17,258 g dry weight ha^−1^ in bryophyte-rich sampling plots and 9,044 g ha^−1^ in lichen-rich plots. These communities are extremely oligotrophic habitats. Väre et al. [20] conducted studies in dry oligotrophic Scots pine forests in Finland in 14 permanent sites during 1991–1994. The proportion of mycorrhizal fungi was 97.6% of the total yield. They used the same method of harvesting, drying, and weighing sporocarps as in our study. The annual yield was 4,600 g dry weight ha^−1^ on average, and the range was 40–18,180 g dry weight ha^−1^. In Finland, in tree stands with pine, the yield was highest in dryish mossy, at the two southernmost study sites, and was lowest in eastern Lapland. The differences were related to the type of vegetation, the yield being lower at dry lichen-rich sites than at mossy sites. This fact is also confirmed by our study. The yield was almost half lower compared to bryophyte-rich plots.

The weather condition (especially rainfall and temperature) was a crucial factor involved in the fungal fructification [66]. The differences in production of biomass were found between lichen- and bryophyte-rich sampling plots in particular months of the study. In areas with large share of lichen, the maximum sporocarp biomass was observed in September, while in bryophyte-rich plots it was observed in October. The subsequent increase in the production of sporocarps in bryophyte-rich sampling plots after intense precipitation in July and August 2021 can be explained by the fact that mosses accumulate large amounts of water [13,31], creating suitable conditions for the development of fungi species that create large and massive sporocarps. Before water reaches the soil, it is stored by bryophytes. On the other hand, in plots rich in lichen, it reaches directly into the soil because lichens do not have the ability to retain water. No significant differences were found between the plots in the fluctuations of the number of taxa in individual months. For both type areas, the highest number of taxa was recorded in October. Our results only partially support the second hypothesis, that there are significant differences in the phenology of ectomycorrhizal fungi between the two types of plots. The differences in production of biomass were found, but there were no significant differences in the fluctuations of the number of taxa between lichen- and bryophyte-rich sampling plots. The phenology of ectomycorrhizal fungi in the lichen-rich sampling plots is consistent with the results of similar studies [41]. The highest abundance of both sporocarps and ectomycorrhizal roots was determined in September, because increased root colonization by ectomycorrhizal fungi in spring and autumn could be associated with a period of intensive root growth and production of sporocarps of ectomycorrhizal fungi. The largest number of species of ectomycorrhizal fungi in the lichen Scots pine forest in Lithuania was found in the autumn period (September and October) [40,41].

### 4.3 Application of the field survey of sporocarps to evaluate the effectiveness of active conservation management – opportunities and limitations

The active protection measures implemented in 2017 in Bory Tucholskie National Park have created the possibility to check how the fungal community in the *Cladonio-Pinetum* habitat will change. Our study is not only the first in terms of distinguishing two stages of preservation of the lichen Scots pine forest habitat but also the first where the fungal communities were studied after the active protection measures, carried out especially for conservation of the habitat.

The study of the response of fungal communities may provide an opportunity to evaluate the efficacy of active protection treatments. Their sporocarps are sensitive to changes in the habitat [20,24,25,26].

The active habitat protection may have an impact on sporocarps biomass production. Influence of thinning and litter removing were the objective of many studies [27]. Differences in fungal productivity have been demonstrated resulting from different management treatments. Both the areas rich in bryophytes (20, 21, and 23) and those rich in lichens (14, 17, and 22) were subjected to thinning to the level of 0.6 (canopy cover – 60%), but differences in biomass production were visible. The biomass was found to be higher in areas with bryophytes. Thinning does not have the same effect on all species [67], they reported that only three ectomycorrhizal species (*Suillus bovinus, Gomphidius roseus,* and *Cortinarius semisanguineus*) out of 19 considered in the study showed an increase in sporocarp production. In our study, the number of sporocarps of *Cortinarius semisanguineus* and biomass in both type of localities were significantly higher in lichen rich areas.

At our lichen-rich sampling plot nos 17 and 22, litter removal was carried out in order to restore oligotrophic conditions favorable to lichens. Although it is difficult to assess the impact of litter raking based on our data, several studies indicate a significant impact of this treatment on fungal communities. Baar and Kuyper [68], and Baar and ter Braak [69] observed that removal of litter and humus layers had a positive effect on the ectomycorrhizal fungi. Sod-cutting had a positive effect on the number of ectomycorrhizal species and on the number of sporocarps. A field experiment conducted in five stands of mature Scots pine in the Netherlands also confirmed previous research results.

Mycologists have long proven that a direct way to gather information about sporocarps biomass of fungi is to collect all of the sporocarps from sampling plots, and then to dry and weigh them [26,29,70,71]. It is not an easy method because it creates many problems, such as rapid autolysis, damage to fragile specimens, interference in the analysis caused by adherent soil particles, and difficulty in collecting and drying specimens separately [26]. Other shortcomings of this method include the fact that the sporocarps often were not representative at the time of harvest – they were too young, which causes an underestimation of the biomass, too mature, which is associated with the colonization of insects, or bitten by other animals. Vogt et al. [65] wrote about such problems. Therefore, the method more often used is to determine the average dry weight of typical, well-developed sporocarps of a given species collected in a herbarium collection (approximately 30 pieces) and multiply it by the number of sporocarps of this species recorded on the observation plot at a given time [72]. Some authors also use special coefficients, such as the conversion rate [32,73] or cap area index [26]. However, the method does not take into account the actual cap sizes of the tested sporocarps but provides data on the potential maximum biomass. Our study needed more accurate data to show the differences between the two types of sampling plots. In this case, there is no probability that the sporocarps will be counted twice. In our study, we tried to verify whether the sporocarps can be used to assess the condition of the lichen Scots pine forests habitat. Of course, the first method of assessment is the degree of coverage of the undergrowth by lichen [18]. It can be used as an additional method that allows for the estimation of the direction of changes in the community and its conservation, because fungi are good bioindicators of changes and are the first to react to them [20,24,25,26,28,32]. Our method can contribute to better protection of these valuable habitats because the inclusion of another group of organisms provides a broader perspective on the condition of the habitat. This method requires long-term studies, which should include both indicator species, as well as protected and rare species [30]. The formation of sporocarps by the red list species [59] in the study area suggests the value of this habitat and the optimal conditions for their formation. Studies using molecular techniques will only show the presence of mycelia in these species and they require higher financial costs.

## 5 Conclusion

In the two types of habitat conservation in the lichen Scots pine forest, differences were found in species richness, composition, and biomass of sporocarps. It seems that the factors that have the strongest influence on fungi in the studied areas (apart from rainfall and temperature) are the type of undergrowth and the age of the forest stand. This is the first time such study has been carried out, but long-term study is needed. Such study has been conducted for 6 years and will be the subject of the article, regarding the factors influencing the distinct composition of mycobiota in two stages of conservation of the lichen Scots pine forest habitat. Our method has not been used to evaluate the condition of these community until now. The study we have conducted shows new possibilities of its application, in addition to the method based on assessing the degree of undergrowth coverage by lichens [18]. Fungi are sensitive bioindicators of changes in the habitat and are often the first to respond to them [20,24,25,26,28,32]. Moreover, the method does not require large financial costs. The extension of the method to include indicator species would allow for the assessment of the habitat by non-mycologists. The presence of several species on the red list [59] proves the value of these areas. The study we have conducted also contributes to understand the biodiversity of these valuable habitats.

## Supplementary Material

Supplementary Table

## Appendix A

**Alphabetical list of fungal taxa identified in sampling plots in 2021**

(Bory Tucholskie National Park, *Cladonio-Pinetum* community, forest compartments 18 and 19)

Abbreviations: V–XI – months of studies, 1–25 – numbers of experimental localities, 26 – control experimental localities, * – species annotated on Red list of the macrofungi in Poland (Wojewoda and Ławynowicz, 2006).

*Amanita excelsa* (Fr.: Fr.) Bertill. f. *excelsa* (= *A. spissa* (Fr.) O. Kumm.) – on soil, among mosses and lichens, under pines; 1, IX-X; 4, 8, 9, 16, IX; 17, VIII; 18, 19, 22, 24, 25, IX.

*Amanita gemmata* (Fr.) Bertill. – on soil, among mosses and lichens, under pines; 1, VII; 3, VII, X; 8, VI, X-XI; 9, 10, 12, 16, 20, X; 22, 24, IX-X; 25, VI.

*Amanita muscaria* (L.: Fr.) Pers. var. *muscaria* – on soil, among mosses, under pines and birches; 4, X; 8, IX-X; 9, IX; 11, 12, X; 13, IX; 14, X; 17, 18, X.

*Amanita rubescens* Pers.: Fr. f. *rubescens –* on soil, among mosses and lichens, under pines; 1, 4, IX-X; 5, 8, IX; 9, IX-X; 10, 12, IX; 15, IX-X; 17, 19, IX; 22, VIII-X; 24, IX; 25, VIII-IX.

*Amanita vaginata* (Bull.: Fr.) Lam. f. *vaginata* – on soil, among mosses, under pines and birches; 4, IX.

*Amaropostia stiptica* (Pers.) B.K. Cui, L.L. Shen & Y.C. Dai – on stump of pine; 25; X-XI.

*Ascocoryne sarcoides* (Jacq.) J.W. Groves & D.E. Wilson – on stump of birch; 4, X, XI.

*Baeospora myosura* (Fr.: Fr.) Singer – on pine cones; 14, X-XI; 21, 24, X.

*Bjerkandera adusta* (Willd.) P. Karst. – on stump of birch; 5, V; 8, IX.

*Boletus edulis* Bull.: Fr. – on soil, among lichens, under pines; 3, VIII-IX; 4, IX; 7, IX-X; 8, VIII-IX; 14, IX-X; 15, X; 16, 17, VIII-X; 18, X; 19, 20, IX; 21, VIII; 26, IX.

*Boletus pinophilus* Pilát & Dermek – on soil, among lichens, under pines; 7, VIII-IX; 12, 14, IX.

*Calocera cornea* (Batsch) Fr.– on stump of birch; 7, VIII, XI; 8, X-XI; 9, X; 10, VII, X; 15, VIII; 19, VII-XI; 21, X; 23, IX-X; 24, X; 25, VII, X-XI.

**Calocera furcata* (Fr.) Fr. – on stump of pine and remains of bark; 1, VIII-XI; 2, VIII-X; 3, VII, IX; 4, X; 5, X-XI, 7, IX-X; 8, VII-XI; 9, X-XI; 10, IX-XI; 11, 13, X; 19, VIII-XI; 21, IX-XI; 25, IX-X; 26, IX.

*Calocera viscosa* (Pers.) Fr. – on dead wood buried in the soil; 3, VIII; 6, 8, 13, X; 26, VIII.

*Cantharellus cibarius* Fr. – on soil, among mosses and lichens, under pines; 3, 4, VIII, X; 5, VIII, X-XI; 7, VII; 8, VII, IX-X; 9, VIII; 10, IX; 11, VIII-X; 12, IX; 13, VIII-X; 14, IX; 15, 18, VIII-IX; 19, VIII; 20, VIII-IX; 21, 23, VIII; 25, IX.

*Chondrostereum purpureum* (Pers.: Fr.) Pouzar – on stump of birch; 4, X-XI; 5, XI.

*Chroogomphus rutilus* (Schaeff.: Fr.) O.K. Mill. var. *rutilus* – on soil, under pine; 4, XI.

*Clitocybe diatreta* (Fr.: Fr.) P. Kumm. – on litter; 1, 24, X.

*Clitocybe metachroa* (Fr.: Fr.) P. Kumm. var. *metachroa* – on litter, among mosses; 24, VIII, X-XI.

*Clitocybe vibecina (Fr.) Quél.* – on needles of pine and remains of bark; 21, XI; 26, X.

*Clitopilus prunulus* (Scop.: Fr.) P. Kumm. – on soil, among mosses; 8, X.

*Collybia cirrhata* (Schumach.) Quél. *(“cirrhata”)* – on decaying sporocarps of other fungi; among mosses; 18, VII; 23, X; 24, XI.

**Coltricia cinnamomea* (Jacq.) Murrill – on soil, among mosses, under pines; 3, 4, IX; 5, VI; 8, VII; 9, VII, IX; 17, X; 22, IX; 25, VII-VIII.

*Coltricia perennis* (L.) Murrill – on soil, under pines; 9, VIII; 25, X.

**Cortinarius acutus* (Pers.) Fr. – on soil, among litter, under pines; 3, X; 4, XI; 7, X; 9, X-XI; 11, 12, X; 14, IX; 15, X; 17, 18, X-XI; 19, XI; 20, X; 22, 23, X-XI; 24, X; 25, XI; 26, X-XI.

*Cortinarius alboviolaceus* (Pers.: Fr.) Fr. – on soil, among mosses and lichens, under birches; 4, X.

**Cortinarius armeniacus* (Schaeff.: Fr.) Fr. *–* on soil, among mosses and lichens, under pines; 4, XI; 7, 13, 14, X; 20, XI; 23, 24, 25, X.

*Cortinarius armillatus* (Fr.: Fr.) Fr. – on soil, among mosses and lichens, under birches; 11, IX-XI.

*Cortinarius brunneus* (Pers.: Fr.) Fr. – on soil, among lichens, under pines; 2, XI; 9, IX; 10, XI; 12, 13, X; 14, IX-X; 15, X-XI; 16, 18, X-XI; 22, 23, X; 24, XI; 25, X.

*Cortinarius caperatus* (Pers.: Fr.) (= *Rozites caperatus* (Pers.: Fr.) P. Karst.) – on soil, among mosses and lichens, under pines; 1, X; 2, IX-X; 3, 4, IX; 6, IX-X; 7, VIII-IX, 12, 15, 18, 20, 23, IX; 24, IX-X.

*Cortinarius cinnamomeus* (L.: Fr.) Gray – on soil, among lichens, under pines; 1, X; 9, IX-X; 12, 18, 21, X; 25, IX.

*Cortinarius comptulus* M.M. Moser – on soil, among mosses and lichens, under pines; 2, 3, 4, 5, 7, 12, X.

**Cortinarius croceus* (Schaeeff.: Fr.) Gray subsp. *croceus* – on soil, among mosses and lichens, under pines and birches; 1, 2, 3, 4, 5, 7, 8, 9, X-XI; 10, XI; 11, X; 12, X-XI; 13, IX-XI; 14, 15, 16, 17, 18, 20, X-XI; 21, XI; 22, 23, 24, X-XI; 25, X; 26, X-XI.

*Cortinarius depressus* Fr. – among lichens and litter, under pines; 20, X.

*Cortinarius glandicolor* (Fr.) Fr. – on soil, among mosses and lichens, under pines; 1, X; 6, IX.

*Cortinarius mucosus* (Bull.: Fr.) J.J. Kickx – on soil, among mosses and lichens, under pines; 1, X; 3, VIII-XI; 4, 5, 7, IX-X; 9, IX; 11, IX-X; 12, 13, IX-X; 14, IX-XI; 15, 16, IX-X; 17, 18, 24, IX; 25, 26, X.

*Cortinarius obtusus* (Fr.: Fr.) Fr. s. lato – on soil, among lichens and litter, under pines; 6, 14, X; 20, XI; 24, X.

*Cortinarius pholideus* (Fr.: Fr.) Fr. – on soil, among lichens, under birches; 11, X-XI.

*Cortinarius pinophilus* Soop – on soil, among lichens, under pines; 4, 26, X.

*Cortinarius quarciticus* H. Lindstr. – on soil, among mosses, under pines; 1, X-XI; 6, 12, 24, X; 26, IX.

*Cortinarius semisanguineus* (Fr.: Fr.) Fr. – on soil, among mosses, under pines; 1, 2, 3, 4, X-XI; 5, X; 6, 9, 11, X-XI; 12, X; 13, 14, 15, 16, X-XI; 17, X; 18, X-XI; 19, X; 20, 21, X-XI; 22, IX-XI; 23, 24, X-XI; 26; IX-XI.

**Cortinarius triumphans* Fr. – on soil, among mosses and lichens, under birches; 4, X.

*Cystoderma amianthinum* (Scop.: Fr.) Fayod – on soil, among mosses; 1, X; 6, 9, XI; 11, 13, X; 20, XI; 21, 24, X; 26, X-XI.

**Dacrymyces chrysospermus* Berk. & M.A. Curtis – on stump of pine; 1, 7, V; 8, V, VIII; 9, VII; 15, V, IX; 17, VII; 18, VI; 19, VII-VIII; 21, X; 22, V-X; X; 26, V.

*Dacrymyces stillatus* Nees – on fallen twigs of pine; 2, V; 3, VII-VIII; 5, V, VII; 7, 13, V; 15, VI; 16, VI-VII; 17, V-VI, X; 19, V, VIII; 21, X-XI; 23, VIII, X.

**Deconica montana* (Pers.: Fr.) P. Kumm. – on mosses (*Polytrichum piliferum*); 14, V, VII, XI; 22, V-VIII, XI.

*Exidia saccharina* Fr. – on stump of pine; 10, VII-VIII; 24, X; 25, VII.

*Fomitopsis betulina* (Bull.) B.K. Cui, M.L. Han & Y.C. Dai (= *Piptoporus betulinus* (Bull.) P. Karst.) – on branch of birch; 4, VI, IX-X.

*Galerina hypnorum* (Schrank : Fr.) Kühner s. Horak 2005, de Haan & Walleyn 2006 – on mosses (*Dicranum* sp., *Pleurozium schreberi*); 1, 2, X-XI; 3, X; 4, X-XI; 5, X; 6, X-XI; 7, XI; 9, X-XI; 11, XI; 12, X-XI; 13, X; 14, 15, X-XI; 16, X; 17, XI; 18, X-XI; 19, XI; 20, VIII, X-XI; 21, X-XI; 22, V, X; 23, 24, X-XI; 26, IX-X.

**Gomphidius roseus* (Fr.: Fr.) P. Karst. – on soil, among mosses, under pines; 5, X; 11, XI; 26, IX-X.

*Gymnopilus decipiens* (W.G. Sm.) P.D. Orton – on dead wood of birch; 2, VII; 4, VII; 9, VII, IX; 13, VII; 16, VII-VIII; 19, VII, IX; 20, VII; 21, IX-X; 23, VII-IX.

*Gymnopilus sapineus* (Fr.: Fr.) Maire s. Kühner & Romagnesi, Moser, Ludwig, Breitenbach & Kränzlin, Horak, non Fries 1821, Hᴓiland 1990 – on stump of pine; 2, VIII, X-XI; 4, VIII; 10, X; 14, VIII, X; 16, VIII-IX; 19, VIII-X; 21, 23, VIII-IX; 25, X.

*Gymnopus androsaceus* (L.: Fr.) J.L. Mata & R.H. Petersen (= *Setulipes androsaceus* (L.: Fr.) Antonín) – on needles of pine, on pine cone; 1, VII-X; 2, VII-IX; 3, VIII; 4, VII-VIII; 5, X; 6, 8, VIII; 9, VII-VIII; 10, VIII; 11, VIII-IX; 13, IX; 15, VIII; 17, VIII, X-XI; 18, VIII-IX; 20, VIII-IX, XI; 21, VIII-XI; 23, VIII, X-XI; 24, VIII, XI; 25, VIII; 26, VIII-IX.

**Gymnopus putillus* (Pers.: Fr.) (Fr.: Fr.) Antonín, Halling & Noordel. – on litter; 1, 2, 3, X-XI; 4, 6, XI; 7, 9, X-XI; 11, XI; 14, X-XI; 15, X; 17, X-XI; 18, X; 20, X-XI; 21, X; 22, 23, 24, 26, X-XI.

*Gyromitra esculenta* (Pers.) Fr. – on soil; 3, V.

*Hypholoma capnoides* (Fr.: Fr.) P. Kumm. – on stump of pine; 13, IX.

*Hypholoma fasciculare* (Huds.: Fr.) P. Kumm. var. *fasciculare* – on stump of pine; 6, X; 11, IX.

*Inocybe sambucina* (Fr.: Fr.) Quél. – on sand, under pines; 13, 17, X.

**Ischnoderma benzoinum* (Wahlenb.) P. Karst. – on stump of pines; 1, 2, X-XI; 3, 4, X; 6, 7, X-XI; 9, X; 13, XI; 18, X; 20, X-XI; 21, X; 25, X.

*Laccaria bicolor* (Maire) P.D. Orton – on soil, among mosses and lichens, under pines; 13, X.

*Laccaria laccata* (Scop.: Fr.) Cooke – on soil, among mosses and lichens, under pines; 2, 3, XI; 4, 5, X-XI; 7, X; 8, XI; 10, 12, 13, X-XI; 14, IX-XI; 15, X; 19, IX-X; 21, 22, 25, X.

*Laccaria proxima* (Boud.) Pat. – on soil, among lichens, under pines; 12, 13, X; 21, XI.

*Lactarius necator* (Bull.: Fr.) Pers. (= *L. turpis* (Weinm.) Fr.) – on soil, among mosses and lichens, under birches; 4, IX-X; 11, IX-XI; 21, IX.

*Lactarius rufus* (Scop.: Fr.) Fr. – on soil, among mosses and lichens, under pines; 1, 3, X; 4, IX-X; 7, X-XI; 9, IX-X; 11, IX; 12, 13, XI; 14, 15, 16, X; 17, 18, IX; 19, X; 20, 21, IX; 22, IX-X; 23, VIII-X; 26, IX.

*Lactarius vietus* (Fr.: Fr.) Fr. – on soil, among mosses, under birches; 4, X.

*Leccinum scabrum* (Bull.: Fr.) Gray – on soil, among lichens, under birches; 4, VI.

*Leccinum versipelle* (Fr. & Hök) Snell – on soil, among lichens, under birches; 4, VIII, X.

*Lyophyllum fumosum* (Pers.: Fr.) P.D. Orton (= *L. conglobatum* (Vitt.) M.M. Moser, *L. decastes* (Fr.: Fr.) Singer var. *fumosum* (Pers.: Fr.) Kühner) – on soil, among mosses and litter; 15, IX.

*Mycena cinerella* (P. Karst.) P. Karst. – on litter; 5, 9, VII; 14, 15, XI; 20, X; 23, 24, X-XI; 26, XI.

*Mycena vulgaris* (Pers.: Fr.) P. Kumm. – on litter, among mosses; 1, VII, IX; 4, XI; 5, IX; 6, VII, IX-X; 7, VIII-IX; 11, VII-VIII; 12, VII; IX; 13, VIII, XI; 14, X; 20, IX; 23, VIII-IX; 24, 26, IX.

**Myxomphalia maura (Fr.: Fr.) Hora* – on soil, among *Polytrichum piliferum*; 22, XI.

*Paxillus involutus* (Batsch: Fr.) Fr. – on soil, among mosses, under pines; 1, 3, X; 4, X-XI; 5, XI; 6, X-XI; 7, X; 9, X-XI; 10, V; 11, IX-X; 12, X; 13, X-XI; 14, X; 18, X-XI; 19, 21, 22, X; 24, IX-XI; 25, 26, X.

*Phlebia tremellosa* (Schrad.) Nakasone & Burds. – on stumps of pines; 1, 2, 3, V-XI; 4, VII-VIII; 5, V-XI; 6, V-IX; 7, V-XI; 8, V-X; 9, V-XI; 10, 11, VI-XI; 12, 13, V-XI; 14, VII-VIII; 15, 16, V-XI; 17, V-VIII; 18, V-VII; 19, 20, 21, V-XI; 22, V-VII; 23, V-XI; 24, V-IX; 25, V-XI.

*Phlebiopsis gigantea* (Fr.) Jülich – on stumps of pines; 1, 2, VI-XI; 7, V-VII; 8, VI-X; 9, IX; 10, V-XI; 13, VI; 14, X-XI; 15, VI-IX; 16, V; 18, V-XI; 21, X-XI; 22, V; 23, V, X; 25, V-VI.

*Pholiota mixta* (Fr.) Kuyper & Tjall.-Beuk. – on soil, among litter; 3, X; 7, 9, IX-X; 14, VIII-XI; 15, X-XI; 16, X; 17, X-XI; 18, 22, 25, IX-XI.

*Pholiotina filipes* (G.F. Atk.) Singer – on litter, among mosses; 1, 4, 6, X; 24, X-XI.

*Rhizopogon luteolus* Fr. & Nordholm (=*R. obtextus* (Sprengel) R. Rauschert; *R. virens* (Alb. & Schwein.) Fr.) – underground, partially underground, under pines; 8, IX.

*Rhodocollybia butyracea* (Bull.: Fr.) Fr.) Lennox f. *butyracea –* on soil, among needles of pines; 1, 6, 13, 19, X; 24, X-XI.

*Russula adusta* (Pers.: Fr.) Fr. – on soil, among mosses and lichens, under pines; 1, 2, IX; 4, VIII-IX 8, IX; 9, IX-X; 10, 15, IX.

*Russula aeruginea* Lindblad – on soil, under birches; 4, VIII.

*Russula betularum* Hora – on soil, under birches; 4, IX, XI.

*Russula decolorans* Fr. – on soil, among mosses, under pines; 1, VII, IX-X; 2, IX; 4, VIII; 6, IX, XI; 7, IX; 11, VIII-IX; 18, IX-X; 20, VII-IX; 22, IX; 23, VIII-IX.

*Russula emetica* (Schaeff.: Fr.) Pers. s. lato – on soil, among mosses, under pines; 1, IX-XI; 2, X-XI; 3, IX-X; 4, VIII, X-XI; 5, 6, 7, IX-XI; 9, IX, XI; 11, X-XI; 12, XI; 13, IX-XI; 14, X; 15, X-XI; 17, IX; 18, IX-XI; 20, IX-X; 21, X-XI; 22, IX-X; 23, 24, IX-XI; 25, IX, XI; 26, IX-XI.

*Russula paludosa* Britzelm – on soil, among mosses, under pines; 1, VIII-X; 2, VIII-IX; 3, IX; 5, 6, 7, VIII-IX; 9, IX; 10, IX-X; 13, VIII-IX; 14, 16, 17, IX; 18, 20, 21, 22, 23; 24, VIII-IX; 25, 26, IX.

*Russula vesca* Fr. – on soil, among mosses, under pines; 2, X; 9, 13, 23, IX.

*Sarcodon squamosus* (Schaeff.) Quél. – on soil, among mosses and lichens, under pines; 1, X-XI; 8, X; 14, XI; 17, 18, 19, 20, X; 24, IX-X; 25, X.

*Stereum hirsutum* (Willd.) Pers. – on stump of birch; 4, VII; 5, IX; 11, IX.

*Stereum rugosum* Pers. – on stump of birch; 5, VI-IX; 11, VIII; 12, V; 13, V-XI.

*Stereum sanguinolentum* (Alb. & Schwein.) Fr. – on dead wood od pines; 5, IX.

*Strobilurus stephanocystis* (Hora) Singer – on cones of pine; 4, 5, 7, V.

*Strobilurus tenacellus* (Pers.: Fr.) Singer – on cones of pine; 7, 17, V.

*Suillus bovinus* (L.: Fr.) Roussel – on soil, among mosses and lichens, under pines; 3, IX-XI; 8, 13, 14, X; 15, X-XI; 22, 24, 26, X.

*Suillus luteus* (L.: Fr.) Roussel – on soil, among mosses and lichens, under pines; 14, IX.

*Suillus variegatus* (Sw.: Fr.) Kuntze – on soil, under pines; 1, X.

*Tapinella atrotomentosa* (Batsch: Fr.) Šutara – at stump od pine; 2, 3, VIII-IX; 6, 8, 9, VIII; 10, IX; 12, VIII; 25, IX-XI.

*Thelephora terrestris* Ehrh. – on soil, under pines; 1, VII, X-XI; 2, X; 3, V-XI; 4, V-VIII; 5, V, IX-XI; 6, V, VIII, X; 7, V-X; 8, V-XI; 9, V-X; 10, VI-X; 11, V-IX; 12, V, X; 13, V, VII-VIII; 14, V-XI; 15, V, IX-XI; 16, V-XI; 17, V, X-XI; 18, V, VIII-IX; 19, V-XI; 20, V-VI, IX-XI; 22, V, VII, IX, XI; 24, V-XI; 25, V-X.

*Trametes ochracea* (Pers.) Gilb. & Ryvarden – at stump of birch; 4, V-X.

*Trametes versicolor* (L.) Lloyd – at stump of birch; 5, VI, VIII-IX; 11, VI-VII; 13, V.

*Trichaptum abietinum* (Pers.) Ryvarden – on stumps of pines; 2, V-VI; 3, IX; 4, 25, V.

*Trichaptum fuscoviolaceum* (Ehrenb.) Ryvarden – on stumps of pines; 2, 9, V; 10, V-XI; 16, V-XI; 20, V-VII, X; 21, V; 25, V-VIII.

*Tricholoma albobrunneum* (Pers.: Fr.) P. Kumm. – on soil, among lichens, under pines; 4, 8, 12, X; 14, IX; 17, 19, X.

**Tricholoma equestre* (L.: Fr.) P. Kumm. (= *T. flavovirens* (Pers.: Fr.) S. Lundell) – on soil, among sand, under pines, among lichens; 1, 2, 3, X-XI; 7, X; 8, IX; 15, X-XI; 16, XI; 17, 19, 24, X; 25, XI.

*Tricholoma saponaceum* (Fr.) P. Kumm. var. *saponaceum* – on soil, among lichens, under pines; 26, VII.

*Tricholoma terreum* (Schaeff.) P. Kumm. – on soil, among sand, under pines, among lichens; 2, XI; 10, X-XI; 11, X; 14, X; 15, IX-XI; 16, X; 17, X-XI; 18, X; 22, 25, X-XI.

*Tricholoma viridilutescens* M.M. Moser – on soil, among lichens, under pines; 24, X.

*Tricholomopsis rutilans* (Schaeff.: Fr.) Singer – at stump of pine; 20, VIII-IX.

*Xerocomus badius* (Fr.: Fr.) E.-J. Gilbert – on soil, among mosses and lichens, under pines; 1, IX-X; 4, 5, IX; 6, X; 8, IX-X; 10, IX; 11, VIII-X; 12, VI, VIII-XI; 13, IX-X; 15, IX, XI; 16, IX-X; 17, IX-XI; 18, XI; 19, IX-X; 20, VIII-X; 21, IX-XI; 23, 24, VIII-XI; 25, IX-X.

## Appendix B

**Table B1:** Summary of biomass productivity and the sum of sporocarps in sampling plots

No.	Name of taxa	Bryophyte-rich plots (numbers: 20, 23, 21)					Lichen-rich plots (numbers: 22, 17, 14)				
		Number of taxa: 27					Number of taxa: 39				
		Dry biomass		Fresh biomass		Number of sporocarps	Dry biomass		Fresh biomass		Number of sporocarps
		G	g/m²	g	g/m²		g	g/m²	g	g/m²	
1.	*Amanita excelsa* f. *excelsa*	0.00	0.00	0.00	0.00	0	3.00	0.01	30.00	0.10	5
2.	*Amanita gemmata*	5.96	0.02	59.60	0.20	5	9.49	0.03	94.90	0.32	9
3.	*Amanita muscaria* var. *muscaria*	0.00	0.00	0.00	0.00	0	3.41	0.01	34.10	0.11	2
4.	*Amanita rubescens* f. *rubescens*	0.00	0.00	0.00	0.00	0	41.39	0.14	413.90	1.38	40
5.	*Boletus edulis*	6.00	0.02	0.60	0.00	3	14.45	0.05	144.50	0.48	7
6.	*Boletus pinophilus*	0.00	0.00	0.00	0.00	0	3.00	0.01	30.00	0.10	1
7.	*Cantharellus cibarius*	6.35	0.02	63.50	0.21	25	0.17	0.00	1.70	0.01	1
8.	*Coltricia cinnamomic*	0.00	0.00	0.00	0.00	0	0.20	0.00	2.00	0.01	5
9.	*Cortinarius acutus*	0.53	0.00	5.30	0.02	25	1.57	0.01	15.70	0.05	52
10.	*Cortinarius armeniacus*	1.54	0.01	15.40	0.05	4	0.11	0.00	1.10	0.00	1
11.	*Cortinarius brunneus*	0.00	0.00	0.00	0.00	0	5.84	0.02	58.40	0.19	26
12.	*Cortinarius caperatus*	27.00	0.09	270.00	0.90	15	0.00	0.00	0.00	0.00	0
13.	*Cortinarius cinnamomeus*	0.31	0.00	3.10	0.01	2	0.00	0.00	0.00	0.00	0
14.	*Cortinarius croceus* subsp. *Croceus*	9.74	0.03	97.40	0.32	112	24.96	0.08	249.60	0.83	257
15.	*Cortinarius depressus*	0.78	0.00	7.80	0.03	5	0.00	0.00	0.00	0.00	0
16.	*Cortinarius mucosus*	12.99	0.04	129.90	0.43	19	0.00	0.00	0.00	0.00	0
17.	*Cortinarius obtusus*	0.20	0.00	2.00	0.01	2	0.13	0.00	1.30	0.00	2
18.	*Cortinarius semisanguineus*	6.48	0.02	64.80	0.22	30	12.35	0.04	123.50	0.41	90
19.	*Cortinarius* sp.1	0.64	0.00	6.40	0.02	6	0.00	0.00	0.00	0.00	0
20.	*Cortinarius* sp.2	0.96	0.00	9.60	0.03	4	0.00	0.00	0.00	0.00	0
21.	*Cortinarius* sp.3	0.44	0.00	4.40	0.01	1	0.00	0.00	0.00	0.00	0
22.	*Cortinarius* sp.4	0.00	0.00	0.00	0.00	0	0.73	0.00	7.30	0.02	5
23.	*Cortinarius* sp.5	0.00	0.00	0.00	0.00	0	1.31	0.00	13.10	0.04	10
24.	*Cortinarius* sp.6	0.00	0.00	0.00	0.00	0	0.32	0.00	3.20	0.01	2
25.	*Cortinarius* sp.7	0.00	0.00	0.00	0.00	0	1.90	0.01	19.00	0.06	12
26.	*Cortinarius* sp.8	0.00	0.00	0.00	0.00	0	0.21	0.00	2.10	0.01	1
27.	*Cortinarius* sp.9	0.00	0.00	0.00	0.00	0	0.69	0.00	6.90	0.02	3
28.	*Cortinarius* sp.10	0.00	0.00	0.00	0.00	0	0.30	0.00	3.00	0.01	2
29.	*Cortinarius* sp.11	0.00	0.00	0.00	0.00	0	0.65	0.00	6.50	0.02	8
30.	*Cortinarius* sp.12	0.00	0.00	0.00	0.00	0	0.16	0.00	1.60	0.01	2
31.	*Inocybe* sp.	0.00	0.00	0.00	0.00	0	0.04	0.00	0.40	0.00	1
32.	*Inocybe sambucina*	0.00	0.00	0.00	0.00	0	0.63	0.00	6.30	0.02	1
33.	*Laccaria laccata*	1.82	0.01	18.20	0.06	2	1.22	0.00	12.20	0.04	13
34.	*Laccaria proxima*	0.66	0.00	6.60	0.02	1	0.00	0.00	0.00	0.00	0
35.	*Lactarius necator*	1.83	0.01	18.30	0.06	2	0.00	0.00	0.00	0.00	0
36.	*Lactarius rufus*	14.21	0.05	142.10	0.47	21	1.77	0.01	17.70	0.06	4
37.	*Paxillus involutus*	6.07	0.02	60.70	0.20	4	9.08	0.03	90.80	0.30	8
38.	*Russula decolorans*	24.00	0.08	240.00	0.80	8	7.64	0.03	76.40	0.25	3
39.	*Russula emetica*	18.06	0.06	180.60	0.60	22	3.35	0.01	33.50	0.11	7
40.	*Russula paludosa*	245.00	0.82	2450.00	8.17	91	32.50	0.11	325.00	1.08	12
41.	*Russula vesca*	10.00	0.03	100.00	0.33	6	0.00	0.00	0.00	0.00	0
42.	*Sarcodon squamosus*	2.34	0.01	23.40	0.08	20	23.00	0.08	230.00	0.77	8
43.	*Suillus bovinus*	0.00	0.00	0.00	0.00	0	1.58	0.01	15.80	0.05	2
44.	*Suillus luteus*	0.00	0.00	0.00	0.00	0	1.40	0.00	14.00	0.05	1
45.	*Thelephora terrestris*	13.61	0.05	136.10	0.45	40	42.60	0.14	426.00	1.42	136
46.	*Tricholoma albobrunneum*	0.00	0.00	0.00	0.00	0	4.21	0.01	42.10	0.14	6
47.	*Tricholoma equestre*	0.00	0.00	0.00	0.00	0	0.58	0.00	5.80	0.02	2
48.	*Tricholoma terreum*	0.00	0.00	0.00	0.00	0	10.51	0.04	105.10	0.35	27
49.	*Xerocomus badius*	100.21	0.33	1002.10	3.34	50	4.86	0.02	48.60	0.16	5
	Total	517.73	1.73	5117.90	17.06	525	271.31	0.90	2713.10	9.04	779
