# Supplementary material for: Macrofungal sporocarp community in the lichen Scots pine forests
Source: Open Life Sci. 2024 Sep 23;19(1):20220973. doi: 10.1515/biol-2022-0973 (PMC11426384; doi:10.1515/biol-2022-0973)
Supplement: Supplementary Table [file biol-2022-0973-sm.pdf]

Supplementary material

Table S1: Geographical coordinates of the experimental plots (WGS 1984)

No of locality	Longitude X	Latitude Y
1	17.54472015	53.84446585
2	17.54374568	53.84452737
3	17.54256091	53.84423509
4	17.54159746	53.8441871
5	17.5403964	53.84417377
6	17.53935655	53.84396903
7	17.5382405	53.84344593
8	17.53810142	53.84407538
9	17.53798418	53.8446124
10	17.53808574	53.84505888
11	17.53936375	53.84520716
12	17.53996799	53.84532416
13	17.54024492	53.84478959
14	17.54117113	53.84474922
15	17.54121905	53.84548911
16	17.542205364	53.84557767
17	17.54234937	53.8452479
18	17.54318906	53.84579587
19	17.54272417	53.8462789
20	17.54123636	53.84674742
21	17.53982247	53.84691343
22	17.54291806	53.84503202
23	17.54469006	53.84557947
24	17.54511142	53.84523337
Control	17.53448545	53.84815921

Table S2: A phytosociological table of lichen pine forest community (the *Cladonio-Pinetum* association) containing the species of vascular plants – p (trees-A, shrub-B and herbal layers-C), as well as bryophytes – b, and lichens – l (field layer – D)

**No of plots**			**9**	**2**	**13**	**15**	**14**	**17**	**22**	**16**	**3**	**4**	**1**	**5**	**7**	**11**	**18**	**12**		**6**	**21**	**19**	**23**	**8**	**20**	**24**	**10**	
Type of community			Lichen-rich																Constancy	Bryophyte-rich								Constancy
Coverage of layer A	Organism group	Layer	92	92	97	94	85	72	76	87	84	88	83	98	96	96	95	96		94	92	98	89	96	96	91	98	
Coverage of layer B			0	0	1	0	0	0	0	0	0	0	15	0	0	1	0	0		0	0	0	0	0	0	1	0	
Coverage of layer C			2	0	3	1	5	11	3	25	5	1	6	21	1	5	1	5		2	1	1	2	1	6	3	3	
Coverage of layer D			100	84	100	100	71	100	100	64	82	100	85	86	43	100	40	95		100	100	100	100	100	81	100	94	
Height of layer A			9.5	8	6	6	5	6	5	6	6	11	8	10	8	6	8	6		15	8	10	13	8	10	10	10	
Height of layer B			0	0	2	0	0	0	0	0	0	0	150	0	0	45	0	0		0	0	0	0	0	0	92	0	
Height of layer C			30	0	30	33	18	22	16	10	18	17	17	26	10	28	32	34		22	10	30	20	20	16	25	38	
Age of tree stands			39	44	19	24	24	38	38	38	38	24	53	19	39	19	44	19		54	38	38	53	39	38	53	39	
Number of vascular plants			3	1	3	2	2	4	4	2	4	3	6	3	2	5	2	5		3	2	2	3	2	3	5	3	
Number of bryophytes			3	6	5	5	2	4	3	5	4	3	3	4	3	2	5	3		2	3	6	4	4	3	5	5	
Number of lichens			10	5	11	8	14	8	13	7	10	7	5	6	5	7	9	8		3	3	1	4	5	7	9	5	
Pinus sylvestris L.	p	A	40	40	60	60	40	40	50	40	40	60	40	50	50	80	60	60	V	60	60	60	60	40	50	70	60	V
Betula pendula L.	p	A	—	—	—	—	—	—	—	—	—	12	—	—	—	—	—	—	I	—	—	—	—	—	—	—	—	—
Alnus glutinosa (L.) Gaertn.	p	B	—	—	1	—	—	—	—	—	—	—	—	—	—	1	—	—	I	—	—	—	—	—	—	—	—	—
Juniperus communis L.	p	B	—	—	—	—	—	—	—	—	—	—	15	—	—	—	—	—	I	—	—	—	—	—	—	1	—	I
Deschampsia flexuosa (L.) Trin.	p	C	1	—	3	1	—	1	1	—	1	1	1	20	—	1	1	2	IV	1	—	1	—	—	1	1	1	IV
Calluna vulgaris (L.) Hull	p	C	1	—	—	—	—	—	1	25	3	—	3	—	—	1	—	1	III	1	1	—	1	1	5	—	1	IV
Corynephorus canescens (L.) P. Beauv.	p	C	—	—	—	—	—	5	1	—	—	—	1	—	—	—	—	—	I	—	—	—	—	—	—	25	—	I
Fagus sylvatica L.	p	C	—	—	—	—	—	—	—	—	1	—	—	—	—	—	—	—	I	—	—	—	—	—	—	—	—	—
Juniperus communis L.	p	C	—	—	—	—	—	—	—	—	—	—	—	—	—	—	—	—	—	—	—	—	1	—	—	—	—	I
Luzula pallescens Sw.	p	C	—	—	—	—	—	—	—	—	—	—	—	—	—	1	—	—	I	—	—	—	—	—	—	—	—	—
Alnus glutinosa (L.) Gaertn.	p	C	—	—	—	—	—	—	—	—	—	—	—	—	—	—	1	—	I	—	—	—	—	—	—	—	—	—
Pinus sylvestris L.	p	C	—	—	—	—	—	—	—	—	—	—	1	—	—	—	—	—	I	—	—	—	—	—	—	1	—	I
Sorbus aucuparia L. em. Hedl.	p	C	—	—	—	—	—	—	—	—	—	—	—	1	—	—	—	1	I	—	—	—	—	—	—	—	—	—
Spergula morisonii Boreau	p	C	—	—	—	—	5	5	—	—	—	—	—	—	—	—	—	—	I	—	—	—	—	—	—	—	—	—

(Continued)

Table S2: *Continued*

No of plots			9	2	13	15	14	17	22	16	3	4	1	5	7	11	18	12		6	21	19	23	8	20	24	10	
Vaccinium myrtillus L.	p	C	—	—	—	—	—	—	—	—	—	—	—	—	1	—	—	—	I	—	—	—	—	—	—	—	—	—
Pleurozium schreberi (Willd. ex Brid.) Mitt.	b	D	3.5	6	0.3	4.5	—	—	—	6.5	6	0.8	—	7.5	1	16	1.3	—	IV	86	100	54	65	68	34	31	17	V
Dicranum polysetum Sw. ex anon.	b	D	—	6.3	2.8	12	—	2.5	4,3	3.8	—	8,8	7.5	15	3.5	—	1	1	IV	16	18	23	25	2.8	15	7.3	1	V
Dicranum scoparium Hedw.	b	D	23	0.3	26	15	6	2.5	11	14	8.3	30	32	44	20	53	26	40	V	—	0.1	5.3	5	18	22	31	12	V
**Dicranum spurium* Hedw.	b	D	—	—	0.1	0.1	—	—	—	0.1	27	—	—	0.1	—	—	—	—	II	—	—	5.8	—	—	—	2.8	—	II
**Ptilidium ciliare* (L.) Hampe	b	D	0.1	14	4	8.8	—	0.1	—	—	—	—	2	—	—	—	5.3	2.3	III	—	—	7.5	4	4.8	—	—	1.5	III
Polytrichum piliferum Hedw.	b	D	—	3.5	—	—	9.3	0.1	8.8	2.5	—	—	—	—	—	—	—	—	II	—	—	—	—	—	—	—	—	—
Hypnum cupressiforme Hedw.	b	D	—	0.1	—	—	—	—	—	—	3.5	—	—	—	—	—	1.5	—	I	—	—	4.3	—	—	—	2.5	32	II
**Cladonia arbuscula* (Wallr.) Flot.	l	D	53	29	6.3	0.8	5.3	0.1	0.1	0.1	18	24	17	3.3	3.5	5.8	53	29	V	0.5	6.3	4	0.1	3.3	6.5	4.8	1.8	V
**Cladonia mitis* Sandst.	l	D	53	29	25	14	17	49	48	15	18	24	18	3.3	3.5	5.8	0.5	15	V	4	0.1	3.3	6.5	4.8	1.8	8.3	6.5	V
**Cladonia gracilis* (L.) Willd.	l	D	47	5.5	22	38	19	49	69	6.3	14	18	11	2.8	3	13	2.5	13	V	—	—	—	1.8	0.3	6	14	2.5	IV
**Cladonia rangiferina* (L.) Weber ex F.H. Wigg.	l	D	0.5	0.1	18	16	0.5	7.8	1	0.8	0.3	13	2	11	5.5	13	0.3	21	V	1.8	1	—	2.5	4.3	0.3	—	—	IV
**Cladonia uncialis* (L.) F.H. Wigg.	l	D	0.8	19	0.5	—	2.3	0.1	3.8	14	0.8	10	12	1.5	6.5	0.8	0.1	—	V	—	0.1	—	—	1.3	—	3.8	1,9	III
Cladonia zopfii Vain.	l	D	—	—	11	9.3	5.8	1.5	0.3	—	—	—	—	—	—	—	0.1	—	II	—	—	—	—	—	0.3	—	—	I
Cladonia phyllophora Hoffm.	l	D	0.1	—	—	—	—	—	—	0.1	1.8	0.3	—	—	—	—	—	0.5	II	—	—	—	—	1	—	3.3	—	II
Cladonia portentosa (Dufour) Coem.	l	D	—	—	—	—	—	—	—	—	—	—	—	0.1	—	0.5	—	1	I	—	—	—	—	—	—	—	—	—
**Cladonia furcata* (Huds.) Schrad.	l	D	—	—	0.3	—	—	—	—	—	—	—	—	—	—	—	—	—	I	—	—	—	4.8	—	—	0.1	—	II
Cladonia deformis (L.) Hoffm.	l	D	—	—	0.3	2.5	0.3	0.3	4.5	0.1	0.3	—	—	—	—	—	0.3	—	III	—	—	—	—	—	0,3	0,8	—	II
Cladonia rei Schaer.	l	D	5.8	—	0.3	1	0.3	0.1	0.3	—	—	—	—	—	—	—	—	0.3	III	—	—	—	—	—	0.8	—	—	I
Cladonia squamosa (Scop.) Hoffm.	l	D	—	—	1	0.8	0.5	—	—	—	—	—	—	0.3	0.3	—	0.1	—	II	0.1	—	—	—	—	—	3	—	II

(Continued)

Table S2: *Continued*

No of plots			9	2	13	15	14	17	22	16	3	4	1	5	7	11	18	12		6	21	19	23	8	20	24	10	
Cladonia pleurota (Flörke) Schaer.	I	D	1	—	0.1	—	—	—	—	—	0.8	0.1	—	—	—	0.5	0.1	0.8	III	—	—	—	—	—	—	—	—	—
Cladonia glauca Flörke	I	D	—	—	—	—	0.3	—	0.5	—	0.5	—	—	—	—	0.5	—	0.3	II	—	—	—	—	—	—	—	2.5	I
Cladonia verticillata (Hoffm.) Schaer.	I	D	—	—	—	0.5	3.8	—	2	0.3	—	—	—	—	—	—	—	—	II	—	—	—	—	—	0.8	1.5	—	II
Cladonia pyxidata (L.) Hoffm.	I	D	—	—	0.8	—	2.5	—	—	—	—	—	—	—	—	—	0.5	—	I	—	—	—	—	—	—	—	—	—
Cladonia subulata (L.) Weber in F, H. Wigg	I	D	—	—	—	—	0.5	—	—	—	0.3	0.8	—	—	—	—	—	—	I	—	—	—	—	—	—	—	—	—
Cladonia cariosa (Ach.) Spreng.	I	D	—	—	—	—	—	—	—	—	—	—	0.5	—	—	—	—	—	I	—	—	—	—	—	—	—	0.1	I
Cladonia coccifera (L.) Willd.	I	D	—	—	—	—	—	—	3.3	—	0.3	—	—	—	—	—	—	—	I	—	—	—	—	—	—	—	—	—
Cladonia floerkeana (Fr.) Flörke	I	D	—	—	—	—	0.3	—	0.5	—	—	—	—	—	—	—	—	—	I	—	—	—	—	—	—	—	—	—
Cladonia crispata (Ach.) Flot.	I	D	—	—	—	—	—	—	—	—	—	—	—	—	—	—	—	—	—	—	—	—	—	—	—	0.3	—	I
Cladonia fimbriata (L.) Fr.	I	D	—	0.1	—	—	—	—	—	—	—	—	—	—	—	—	—	—	I	—	—	—	—	—	—	—	—	—
Cladonia macilenta Hoffm.	I	D	0.1	—	—	—	—	—	—	—	—	—	—	—	—	—	—	—	I	—	—	—	—	—	—	—	—	—
Cetraria aculeata (Schreb.) Fr.	I	D	0.1	—	—	—	1.8	0.1	5.3	—	—	—	—	—	—	—	—	—	II	—	—	—	—	—	—	—	—	—
Stereocaulon pileatum Ach.	I	D	—	—	—	—	—	—	1	—	—	—	—	—	—	—	—	—	I	—	—	—	—	—	—	—	—	—
Trapeliopsis granusosa (Hoffm.) Lumbsch, in Hertel	I	D	0.1	—	—	—	—	—	—	—	—	—	—	—	—	—	—	—	I	—	—	—	—	—	—	—	—	—

The plots in the table are arranged according to the results of the dendrogram classification (Fig. 6). Coverage for each species are given in percentages. Constancy: I – percentage frequency of 1–20%, II – 21–40%, III – 41–60%, IV – 61–80%, V – 81–100%. Characteristic lichens and bryophytes species for the *Cladonio–Pinetum* association were marked by Asterisk symbol.
